# Targeting *Echinococcus multilocularis* Stem Cells by Inhibition of the Polo-Like Kinase EmPlk1

**DOI:** 10.1371/journal.pntd.0002870

**Published:** 2014-06-05

**Authors:** Andreas Schubert, Uriel Koziol, Katia Cailliau, Mathieu Vanderstraete, Colette Dissous, Klaus Brehm

**Affiliations:** 1 University of Würzburg, Institute of Hygiene and Microbiology, Würzburg, Germany; 2 Universidad de la República, Facultad de Ciencias, Sección Bioquímica y Biología Molecular, Montevideo, Uruguay; 3 EA4479, IFR147, Laboratoire de Régulation des Signaux de Division, SN3, University Lille 1, Villeneuve d'Ascq, France; 4 Center for Infection and Immunology of Lille, Inserm U1019, CNRS-UMR 8204, University Lille 2, Institut Pasteur de Lille, Lille, France; Center for Discovery and Innovation in Parasitic Diseases, United States of America

## Abstract

**Background:**

Alveolar echinococcosis (AE) is a life-threatening disease caused by larvae of the fox-tapeworm *Echinococcus multilocularis*. Crucial to AE pathology is continuous infiltrative growth of the parasite's metacestode stage, which is driven by a population of somatic stem cells, called germinative cells. Current anti-AE chemotherapy using benzimidazoles is ineffective in eliminating the germinative cell population, thus leading to remission of parasite growth upon therapy discontinuation.

**Methodology/Principal findings:**

We herein describe the characterization of EmPlk1, encoded by the gene *emplk1*, which displays significant homologies to members of the Plk1 sub-family of Polo-like kinases that regulate mitosis in eukaryotic cells. We demonstrate germinative cell-specific expression of *emplk1* by RT-PCR, transcriptomics, and *in situ* hybridization. We also show that EmPlk1 can induce germinal vesicle breakdown when heterologously expressed in *Xenopus* oocytes, indicating that it is an active kinase. This activity was significantly suppressed in presence of BI 2536, a Plk1 inhibitor that has been tested in clinical trials against cancer. Addition of BI 2536 at concentrations as low as 20 nM significantly blocked the formation of metacestode vesicles from cultivated *Echinococcus* germinative cells. Furthermore, low concentrations of BI 2536 eliminated the germinative cell population from mature metacestode vesicles *in vitro*, yielding parasite tissue that was no longer capable of proliferation.

**Conclusions/Significance:**

We conclude that BI 2536 effectively inactivates *E. multilocularis* germinative cells in parasite larvae *in vitro* by direct inhibition of EmPlk1, thus inducing mitotic arrest and germinative cell killing. Since germinative cells are decisive for parasite proliferation and metastasis formation within the host, BI 2536 and related compounds are very promising compounds to complement benzimidazoles in AE chemotherapy.

## Author Summary

The lethal disease AE is characterized by continuous and infiltrative growth of the metacestode larva of the tapeworm *E. multilocularis* within host organs. This cancer-like progression is exclusively driven by a population of parasite stem cells (germinative cells) that have to be eliminated for an effective cure of the disease. Current treatment options, using benzimidazoles, are parasitostatic only, and thus obviously not effective in germinative cell killing. We herein describe a novel, druggable parasite enzyme, EmPlk1, that specifically regulates germinative cell proliferation. We show that a compound, BI 2536, originally designed to inhibit the human ortholog of EmPlk1, can also inhibit the parasite protein at low doses. Furthermore, low doses of BI 2536 eliminated germinative cells from *Echinococcus* larvae *in vitro* and prevented parasite growth and development. We propose that BI 2536 and related compounds are promising drugs to complement current benzimidazole treatment for achieving parasite killing.

## Introduction

The metacestode larval stage of the fox-tapeworm *E. multilocularis* is the causative agent of alveolar echinococcosis (AE), a life-threatening zoonosis prevalent in the Northern Hemisphere [1], [2]. Infection of the mammalian intermediate host (rodents, humans) is initiated by oral uptake of ‘infectious eggs’, which contain the oncosphere larva. Upon hatching from the egg in the host intestine, the oncosphere penetrates the intestinal epithelium and gains access to the host organs. Typically within the liver, the parasite then undergoes a developmental transition towards the metacestode stage which is entirely driven by parasite stem cells (germinative cells) that have been carried to the host by the oncosphere [2]–[4]. As an asexual multiplication stage, the metacestode tissue grows multivesicularly and infiltratively, like a malignant tumor, into the surrounding host tissue, eventually leading to organ failure [1]–[4]. In natural rodent infections, head regions of the future adult worm (protoscoleces) are formed from germinative cells of the cellular layer (germinal layer; GL) of the metacestode, and are subsequently taken up when the definitive host takes its prey [4]. In human infections, asexual parasite growth occurs similar to the situation in rodents, but protoscoleces are only formed in rare cases [1]. Human AE is very difficult to treat and only in ∼20% of cases the parasite mass can be removed by surgery [1], [2], [5]. In all other cases, chemotherapy is the only option of treatment and is currently mainly carried out using benzimidazoles, which target parasite β-tubulin [5]. However, β-tubulins between parasite and host are highly similar [6], [7], so that only parasitostatic concentrations of these drugs can be applied to prevent significant adverse side effects [1], [5], [8]. Hence, although the introduction of benzimidazole chemotherapy in the 1990s has significantly improved patient's prognosis, treatment has to be applied for years to decades, and in many cases even life-long [1], [5], [8]. Furthermore, a significant number of patients cannot tolerate benzimidazole treatment at all [1], [5]. Several attempts to improve anti-AE chemotherapy by identifying novel anti-parasitic drugs are currently undertaken [8], [9] but, so far, no reliable alternative to benzimidazole treatment is available. This underscores an urgent need for novel chemotherapeutic options against AE.

A hallmark of both free-living and parasitic flatworms is the employment of a population of totipotent stem cells (called ‘neoblasts’ in free-living species) that decisively contribute to the enormous regenerative capacity and developmental plasticity of this group of organisms [4], [10]. In *Echinococcus*, earlier ultrastructural studies also strongly suggested the presence of undifferentiated stem cells (‘germinative cells’) in parasite larvae and is has been hypothesized that they might contribute to parasite growth [11], [12]. By establishing cultivation techniques for germinative cells, we later demonstrated their proliferative potential and showed that they can form mature metacestode vesicles *in vitro* [13]. Very recently, we also demonstrated that germinative cells are the only proliferative cells in parasite larvae, that they give rise to all differentiated cells, and that there are important differences between the stem cell populations of *E. multilocularis* to those of the related schistosomes, and to neoblasts of free-living flatworms [14]. Since the germinative cells are absolutely decisive for asexual multiplication of the *E. multilocularis* metacestode, they constitute one of the most important cell types for the development of chemotherapeutics to prevent parasite proliferation.

Polo-like kinases (PLKs) are serine/threonine kinases (STK) that act as important regulators of cell-cycle progression in all eukaryotic lineages [15]–[17]. They are particularly important in the M-phase during which they regulate the assembly of the spindle apparatus and the activation of cyclin-dependent protein kinases (CDC) [15]–[17]. In humans, five PLKs are expressed of which Plk1-3 are very similar in structure. They comprise a conserved N-terminal STK domain, necessary for phosphorylation of downstream molecules, and two C-terminal Polo-box domains (PBD), which govern protein-protein-interaction and subcellular localization [15]–[17]. The so far best investigated PLK is mammalian Plk1, which is mainly expressed in late G2 and M phases and regulates both mitosis and meiosis [15], [16]. Most importantly, Plk1 activates the dual-specific phosphatase Cdc25C, which dephosphorylates, and thus activates, the maturation promoting factor (MPF), resulting in nuclear MPF translocation. Since Plk1 is highly expressed in proliferating cells, including many cancer cells, it has already been intensely validated as an anti-cancer drug target, and several inhibitors that specifically inhibit Plk1 activity are available [15], [18].

In contrast to Plk1, Plk2 and Plk3 are encoded by early response genes that are activated during serum stimulation of cells, and both proteins are involved in checkpoint-mediated cell cycle arrest [15]. Plk4 is a divergent member of the PLK family and differs from Plk1-3 in its domain composition. Plk4 shares little overall homology with Plk1-3, and only contains one PBD [15]. Plk4 gene expression increases from late G1 to S phase, and the protein is known to be involved in centriole duplication, and most probably also plays a role in chromosome maturation and mitotic progression [15]. Recently, a fifth PLK was identified in mammals, Plk5, which is localized to the nucleolus and whose expression is induced by stress conditions and DNA damage [15].

PLKs with structural and functional homologies to mammalian Plk1 and Plk4 have already been described in invertebrate model systems such as *Drosophila melanogaster* and *Caenorhabditis elegans* [17]. In free-living flatworms, functional studies on Polo-like kinases have not yet been carried out, although their transcripts have been specifically detected in neoblasts and reproductive organs of the planarian *Schmidtea mediterranea* [19], [20]. In parasitic flatworms, PLKs have so far exclusively been investigated in the trematode *Schistosoma mansoni* [17], [21], [22]. In a first study, Long et al. [21] characterized SmPlk1, which displays considerable homologies to mammalian Plk1, and demonstrated expression of the respective gene in female vitelline cells and oocytes as well as in male spermatocytes, indicating a role of SmPlk1 in schistosome mitosis and/or meiosis. Interestingly, the PLK inhibitor BI 2536, which was originally designed to inhibit human Plk1 [23], induced dramatic alterations in schistosome gonads *in vitro*, which affected oogenesis and spermatogenesis at 100 nM concentrations [21]. Very recently, the same authors also characterized a Plk4-like PLK in *S. mansoni*, named SmSak, which was mostly expressed in schistosome female ovary and vitellarium, and which interacted with SmPlk1 [22]. In contrast to SmPlk1, however, the activity of recombinantly expressed SmSak was not affected by Bi 2536, indicating a high selectivity of this inhibitor for Plk1-like kinases [17], [22].

The successful identification of SmPlk1 in proliferative cells of *S. mansoni*, and its inhibition by an available small molecule compound, prompted us to investigate the role of PLKs in asexual growth of *E. multilocularis* larvae. We herein describe the characterization of a Plk1-like *Echinococcus* kinase, EmPlk1, and demonstrate its expression in germinative cells of the *Echinococcus* larval stages. We show that the activity of EmPlk1, when heterologously expressed in the *Xenopus* oocyte system, can be seriously affected by available PLK inhibitors. Furthermore, using *in vitro* systems for the cultivation of parasite stem cells [13], [24] and metacestode larvae [25], [26] we show that the compound BI 2536 significantly inhibits parasite development already at concentrations as low as 20 nM. The potential of PLK inhibitors in anti AE chemotherapy is discussed.

## Materials and Methods

### Ethics statement

*In vivo* propagation of parasite material was performed in mongolian jirds (*Meriones unguiculatus*), which were raised and housed at the local animal facility of the Institute of Hygiene and Microbiology, University of Würzburg. This study was performed in strict accordance with German (*Deutsches Tierschutzgesetz,TierSchG*, version from Dec-9-2010) and European (European directive 2010/63/EU) regulations on the protection of animals. The protocol was approved by the Ethics Committee of the Government of Lower Franconia (Regierung von Unterfranken) under permit number 55.2-2531.01-31/10.

### Chemicals and inhibitors

PLK inhibitors BI 2536 [23] and BI 6727 (Volasertib) [27] were purchased from Axon Medchem (Groningen, The Netherlands) and Selleckchem.com (München, Germany), respectively. Both inhibitors were dissolved in dimethyl sulfoxide (DMSO) from Sigma Aldrich (D8418-50ML) as 10 mM stock solutions and were stored at −80°C until use (according to the manufacturer's instructions).

### Parasite cultivation and inhibitor tests

All experiments were performed using the *E. multilocularis* isolates H95 (cloning procedures; drug treatment) [28] and GH09 (whole mount in situ hybridization) [28]. Mongolian jirds (*M. unguiculatus*) were used for *in vivo* propagation of the parasite by intraperitoneal passages as previously described [26]. Co-cultivation of metacestode vesicles with host cells was carried out essentially as previously described [26]. Axenic cultivation of metacestode vesicles was performed as described by Spiliotis et al. [25]. *Echinococcus* primary cells were isolated and cultivated under axenic conditions as described by Spiliotis et al. [13]. Conditioned medium (A4 medium) was prepared by seeding 1×10^6^ rat Reuber hepatoma cells [26] together with 100 ml DMEM medium (Life technologies) in a culture flask, followed by 1 week incubation. Subsequently, the supernatant was removed and sterile filtrated (A4 medium). Inhibitor tests on metacestode vesicles were performed under axenic culture conditions (nitrogen atmosphere [13]) in A4 medium as previously described by Hemer *et al.* [29] and Gelmedin *et al.* [30], with up to ten vesicles per well of a 6-well (5 ml vol. per well) culture plate. Primary cell cultures were set up essentially as described by Spiliotis et al. [24] and the amount of isolated cells was subsequently measured indirectly through densitometry. 1 Unit of primary cells was defined as the amount that yields an OD_600_ of 0.02 in Phosphate Buffered Saline (PBS). 50 Units isolated primary cells (∼15.000 cells) were then seeded in a 48-well plate with 1.3 ml A4-medium. Medium was changed every second day and fresh inhibitor was added. Inhibitors were used in final concentrations of 5, 10, 25, 50, and 100 nM. All experiments were performed with at least three technical and biological replicates. Linear regression analysis was carried out using MicrosoftExcel-2007. Error bars in figures represent standard deviation. Differences were considered significant for p values below 0.05 (indicated by (*)), for p between 0.001 and 0.01 (**), and for p<0.001 (***). For p>0.5, differences were considered non-significant.

### Labeling of proliferating cells in metacestode vesicles by EdU pulse staining

Proliferation of stem cells was investigated by staining of newly synthesized DNA using the Click-iT EdU cell proliferation assay (Invitrogen, C10337). Metacestode vesicles from hepatocyte co-culture [26] were washed once with 1× PBS and carefully transferred into a 15 ml tube containing 3 ml A4-medium. A final concentration of 50 µM EdU (Component A) was added and the metacestode vesicles were incubated for 5 h at 37°C (with gentle agitation every 45 min). For fixation, metacestode vesicles were transferred to a Petri-dish and carefully opened with a syringe tip to remove hydatid fluid. After intense washing with 1× PBS, fixative (4% paraformaldehyde (PFA) in 1× PBS) was added and samples were incubated for 1 h at room temperature. The samples were washed again (1× PBS) to remove the fixative, and then transferred to a 1.5 ml tube. The EdU labeling procedure was then carried out according to the manufacturer's instructions (Invitrogen, C10337), but with extended incubation times. Metacestode vesicles were finally analyzed by fluorescence microscopy.

For long-term treatment (21 days) with BI 2536, metacestode vesicles were cultivated in A4-medium for 21 days with inhibitor (medium and inhibitor change every second day), followed by three days recovery without treatment. As a control, medium with and identical amount of DMSO (without inhibitor) was used. Randomly chosen metacestode vesicles (t = 4) were isolated and EdU stained as outlined above.

Of each metacestode vesicle, three randomly chosen sections of the GL were analyzed by microscopy and EdU positive cells were counted. The number of EdU positive cells was calculated to cells per mm^2^ of the GL.

For short-time inhibitor experiments, metacestode vesicles were treated *in vitro* for 24, 48 and 72 hours with 50 nM or 100 nM BI 2536, followed by 24 h regeneration without inhibitor. EdU staining and calculation of Edu positive cells per mm^2^ of GL were subsequently performed as described above.

### Identification and cloning of *emplk1*

The *E. multilocularis* genome sequence assembly [28] was used as available in GeneDB under http://www.genedb.org/Homepage/Emultilocularis. Published sequences of human Plk1 (GenBank accession number: P53350) and SmPlk1 (AY747306) were employed in extensive BLASTP searches on the genome sequence. Amino acid sequence and domain predictions were carried out using UniProt (http://www.uniprot.org/) and SMART (http://smart.embl-heidelberg.de/) software. Based on the genomic sequence information, primers were designed to amplify the full EmPlk1 coding sequence. Total RNA was isolated from metacestode vesicles with TRIzol reagent and reverse transcribed into cDNA as previously described [30]. The cDNA then served as a template for gene amplification by PCR using primers AKO-053 (5′-GAC TTC TGC CCG GGT ATG GAT A-3′) and AKO-054 (5′-GGA AGA CGG CAA ACA TGT GAT-3′). The PCR product was subsequently cloned into pJET 1.2 (Thermo Scientific CloneJET PCR Cloning Kit) and sequenced.

### Expression of EmPlk1 in *Xenopus* oocytes

According to the previously described protocol for heterologous expression of SmPlk1 in *Xenopus* oocytes [21], mutated versions of EmPlk1 were produced which included a constitutively active form of the kinase (T_179_D), a version that cannot be phosphorylated at T_179_ (T_179_V), and kinase dead versions of wild-type EmPlk1 (wt^KD^) and T_179_D (T_179_D^KD^). The kinase dead versions were generated by replacing the highly conserved active loop motif D_163_FG for D_163_SV. Kinase domain mutants were generated by mutagenesis PCR using the following primer combinations: T_179_D (AKO-138, 5′-GGT GAA ATG AAG AAG GAC TTA TGT GGG ACG CCA AAC TAT ATT GCT CC-3′ and AKO-139, 5′-CCA CAT AAG TCC TTC TTC ATT TCA CCT TCT TTA GTA ATT CTA G-3′), T_179_V (AKO-140, 5′-GGT GAA ATG AAG AAG GTA TTA TGT GGG ACG CCA AAC TAT ATT GCT CC-3′ and AKO-141, 5′-CCA CAT AAT ACC TTC TTC ATT TCA CCT TCT TTA GTA ATT CTA G-3′), and D_163_SV (AKO-146, 5′-GAC ATG ATT GTA AAG ATC GGG GAT TCG GTG TTG GCC TCT AGA ATT ACT AAA GAA GG-3′ and AKO-147, 5′-GTA ATT CTA GAG GCC AAC ACC GAA TCC CCG ATC TTT ACA ATC ATG TCA TCA TTT AAA AAC AGA TTG GC-3′). For mutagenesis, the *emplk1* reading frame was cloned into the pBAD TOPO/Thio expression vector (Life Technologies). Mutations were then introduced, employing the above listed primers, using the QuickChange Site-directed mutagenesis kit (Agilent Technologies) according to the manufacturer's instructions. All plasmid constructs were finally sequenced to verify wild-type sequences and the successful introduction of the desired mutation. Wild-type and mutant versions of the EmPlk1 reading frame were further cloned into the pSecTag2/Hygro A vector (Life Technologies) that contains a T7 promoter sequence. cRNA encoding EmPlk1 proteins was synthesized *in vitro* using the T7 mMessage mMachine Kit (Ambion, USA) and injected in stage VI *Xenopus laevis* oocytes according to [31].

Heterologous expression of EmPlk1 wild-type and mutant forms in *Xenopus* oocytes, *in vitro* treatment of oocytes with the inhibitors BI2536 and BI6727, and germinal vesicle breakdown assays were conducted essentially as previously described by Long *et al.* [21] for SmPlk1. As a positive control for GVBD, oocytes were stimulated with progesterone (PG), the natural inducer. All experiments were carried out on samples composed of 20 oocytes originated from three different *Xenopus* females.

### Semi-quantitative RT-PCR

Total RNA was isolated using TRIzol as previously described [32] from 2, 5, and 11 day old primary cell cultures, from dormant and pepsin/low pH activated protoscoleces [33], and from mature metacestode vesicles. Isolated RNA was DNase treated (RQ1 RNase-free DNase, Promega) followed by phenol/chloroform extraction. RNA concentration was quantified by spectrophotometry and 750 ng of each stage were used for reverse transcription (RT) as previously described [32]. Intron-flanking, gene specific primers for *emplk1* (AKO-57; 5′-GAG CAT GTT CAG TGT GAT GG-3′ and AKO-60; 5′-CGA TCT ATC ATA TCG TAG GCG-3′) were used to estimate the semi-quantitative expression profile by PCR employing a protocol of 30 cycles of 30 sec at 94°C, 30 sec at 58°C, and 1 min at 72°C. The constitutively expressed gene *elp* [34] served as a control.

### Whole mount *in situ* hybridization (WISH) of metacestode vesicles

Metacestode vesicles of isolate GHO9 with developing protoscoleces were used for *in situ* hybridization according to Koziol et al. [14]. A 1.3 kb fragment of the *emplk1* open reading frame was amplified and subcloned into pDrive (Qiagen) using primers AKO-55 (5′-CTC TCA TGG AAC TGC ATA AGA G-3′) and AKO-60 (5′-CGA TCT ATC ATA TCG ATG GCG-3′). Digoxygenin-labelled antisense and sense RNA probes were *in vitro* transcribed using the T7 or SP6 promoter of the linearized vector as previously described [14]. Detection was performed using anti-digoxygenin antibodies coupled to alkaline phosphatase, and NBT/BCIP as colorimetric substrates, as described [14].

### Nucleotide sequence accession number

The complete *emplk1* cDNA sequence reported in this paper was submitted to the GenBank database and is available under accession number HG931729.

## Results

### Cloning and characterization of the *emplk1* cDNA

**Figure 1:**
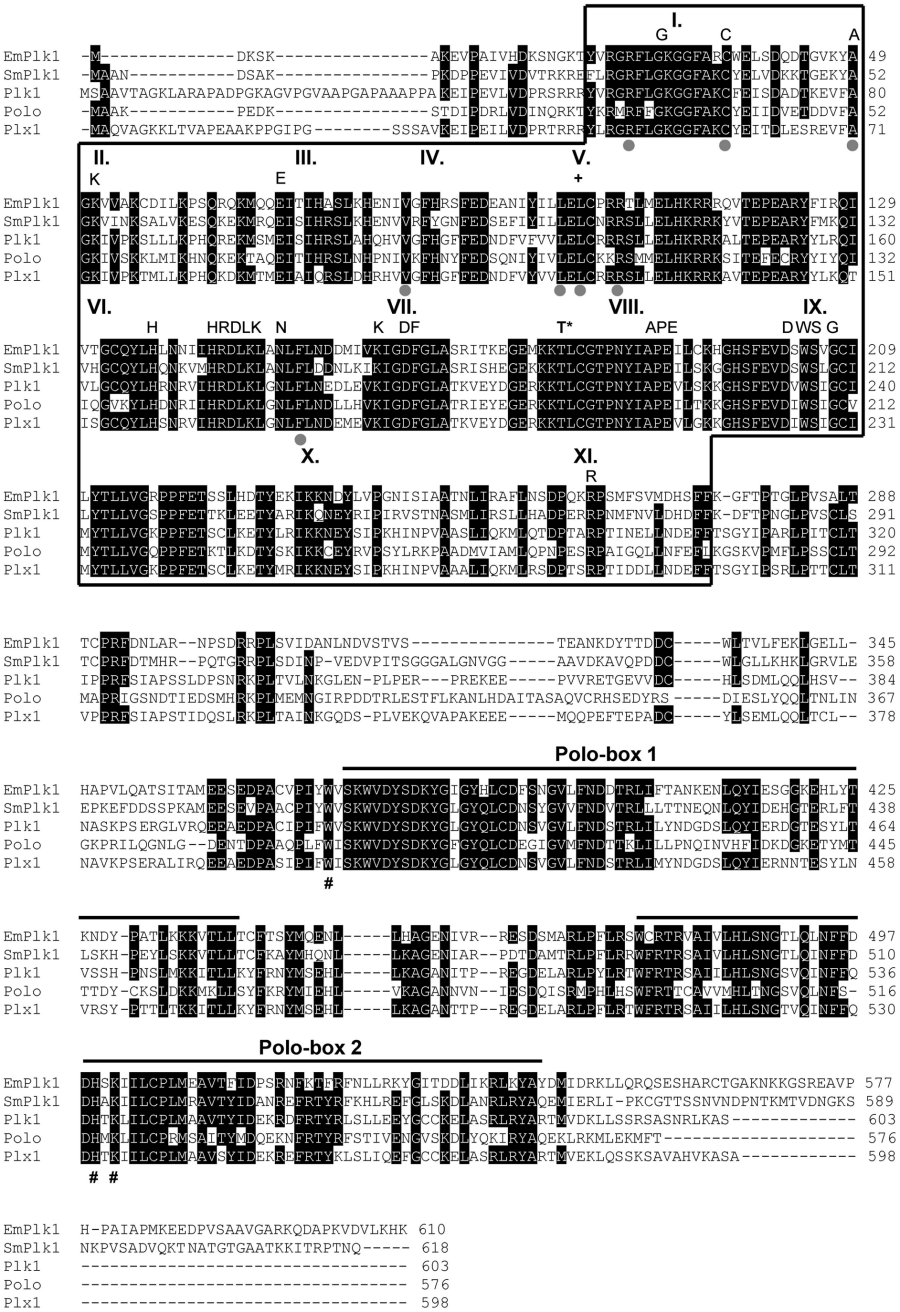
Amino acid sequence and structural analysis of EmPlk1. The EmPlk1 amino acid sequence was aligned to Plk1-like enzymes of *S. mansoni* (SmPlk1; GenBank accession number: AY747306), *Homo sapiens* (Plk1; P53350); *D. melanogaster* (Polo; P52304) and *X. laevis* (Plx1; P70032). The predicted kinase domain is boxed, predicted Polo-boxes (Polo-box 1; Polo-box 2) are indicated above the alignment. Residues identical in at least four of the sequences are shown in white on black background. The eleven sub-domains known to be highly conserved in kinases are indicated above (I–XI). Highly conserved amino acid residues and sequence motifs are indicated above the alignment. The highly conserved threonine residue which is critical for activation is marked by (T*) above the alignment. Residues important for phosphosubstrate binding of the C-terminal domain are marked by (#) below the alignment. Residues involved in binding of BI 2536 to Plk1 are indicated by grey dots below the alignment.

By BLASTP genome mining of the available *E. multilocularis* genome sequence [28] (http://www.genedb.org/Homepage/Emultilocularis) using the full length sequences of human Plk1 [35] and *S. mansoni* SmPlk1 [21] as queries, one single locus encoding an *Echinococcus* Plk1 ortholog (EmuJ_000471700) was identified on chromosome 3. Due to its homologies (see below), the respective gene was designated *emplk1* (*E. multilocularis* Polo-like kinase 1) encoding the protein EmPlk1. The *emplk1* gene spanned a genomic region of 3.174 bp and, like the SmPlk1 encoding gene of *S. mansoni* [21], comprised 7 exons, separated by 6 introns. All introns displayed canonical GT-AG dinucleotide sequences at the 5′ splice donor and the 3′ splice acceptor sites. Informed by the *emplk1* genomic sequence, primers were designed to PCR-amplify the entire *emplk1* reading frame from metacestode cDNA preparations. The cDNA fragment was cloned and fully sequenced, which confirmed the exonic gene sequence as determined by the *E. multilocularis* genome project. The full length EmPlk1 reading frame comprised 1833 bp and coded for a protein of 610 amino acids, EmPlk1, with a calculated molecular mass of 69.5 kDa. Analysis of the EmPlk1 protein sequence by SMART [36] identified an N-terminal STK catalytical domain (between Y_22_ and F_274_) and a C-terminal protein binding domain, which included two Polo box domains (Figure 1). The EmPlk1 kinase domain displayed sequence motifs characteristic of all 11 typical sub-domains (I–XI) of protein kinases and included all residues previously determined to be invariable for protein kinases [37] at the respective positions (Figure 1). Particularly the sequence motifs HRDLKxxN (sub-domain VI) and GTPNYIAPE (VIII) are strong indicators for STK activity [37]. In human Plk1, residue T_210_ was previously shown to be the major phosphorylation site of mitotic PLKs [15] and a respective threonine residue was also conserved in schistosome SmPlk1 (T_182_) [21]. In EmPlk1, this residue was also conserved (T_179_; Figure 1). As in SmPlk1 [21] and human Plk1 [35], the ATP binding site of EmPlk1 contained a GxGGFAxC motif (Figure 1), which is a PLK-typical variation of the canonical GxGxxGxV motif found in the majority of protein kinases [37]. Overall, the EmPlk1 kinase domain displayed significant homologies to the kinase domains of well investigated PLKs such as *S. mansoni* SmPlk1 (68% identical residues), human Plk1 (62%), *Drosophila* Polo (60%), and *Xenopus* Plx1 (60%).

Protein-protein interaction and cellular localization of PLKs is regulated by the C-terminally located protein binding domain [15]. As typical for Plk1-like PLKs, the EmPlk1 protein binding domain contains two Polo boxes (Y_378_-T_441_ and W_475_-Y_545_), which are separated from the kinase domain by a non-conserved linker region. In human Plk1, three residues (W_414_, H_538_, K_540_) have previously been shown to be essential for the binding to phospho-S/T binding motifs, and all three residues are also perfectly conserved in EmPlk1 (W_371_, H_499_, K_501_; Figure 1).

Kothe et al. [38] previously identified several amino acid residues that are important for binding of the PLK inhibitor BI 2536 to human Plk1. In particular, the absence of a bulky side chain at position 132 (L_132_ in human Plk1) was an important specificity determinant that ensured optimal binding of BI 2536 to the Plk1 subfamily of PLKs [38]. In EmPlk1, all these residues, including the leucine residue (L_101_), were conserved at the respective positions (Figure 1).

Taken together, all above analyses clearly identified EmPlk1 as a member of the Plk1 subfamily of PLKs, indicated that the *Echinococcus* protein is most probably enzymatically active, and that the PLK inhibitor BI 2536 should be able to bind to the parasite-derived kinase.

### Expression of *emplk1* in *E. multilocularis* larval stages

**Figure 2:**
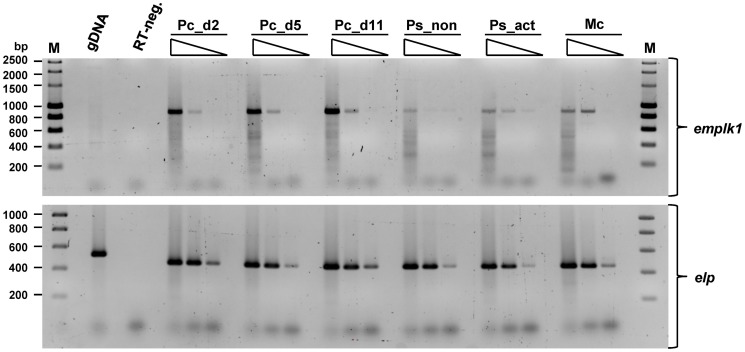
Expression of *emplk1* in *E. multilocularis* larval stages. Total RNA was isolated from primary cell cultures (Pc) after 2, 5 and 11 days (d2, d5, d11) of development towards the metacestode stage, from dormant (Ps_non) and pepsin/low pH-activated protoscoleces (Ps_act), as well as from mature metacestode vesicles (Mc). The isolated RNA was reverse transcribed to cDNA and equal amounts of serial (10-fold) dilutions of cDNA were subjected to gene-specific RT-PCR for *emplk1* and the constitutively expressed gene *elp* (control) as indicated to the right. PCR products were separated on a 1.5% agarose gel and stained with ethidium bromide. gDNA indicates positive control lanes where PCR has been performed on genomic DNA. ‘RT-neg’ indicates the negative control where reverse transcriptase has been omitted. Marker sizes are indicated to the left.

To determine whether *emplk1* is expressed in *Echinococcus* larval stages relevant to the infection of the intermediate host, semi-quantitative RT-PCR experiments were carried out. As relevant parasite stages, we chose mature metacestode vesicles and protoscoleces before and after activation by pepsin/low pH (mimics transition into the definitive host), to cover late stages of the infection. In our established primary cell cultivation system [13], [24], *E.multilocularis* germinative cells are capable of developing into metacestode vesicles similar to the oncosphere metacestode transition process [7]. Hence, in order to cover early stages of the infection, we also included primary cell cultures at different time points (2, 5, 11 days) of development. Total RNA was isolated and, after cDNA preparation, *emplk1* gene specific PCR was carried out on serial dilutions. As shown in Figure 2, *emplk1* transcripts could be clearly identified in all larval stages tested, and particularly prominent expression was observed in primary cell cultures, which typically contain large proportions of parasite stem cells [13], [14].

During the *E. multilocularis* genome project, preliminary deep sequencing transcriptome data were generated for primary cell cultures (2 and 11 days old) as well as for metacestode vesicles and activated/dormant protoscoleces [28]. When we analyzed these profiles we found particularly high expression in primary cell cultures after 2 days of cultivation, which was reduced in primary cells after 11 days, and basal in metacestode vesicles and protoscoleces (Figure S1). This verified the RT-PCR data mentioned above and, again, indicated possible stem cell specific expression of *emplk1* since young primary cell cultures contain particularly high percentages of germinative cells, which steadily decline during development (differentiation) into mature vesicles [14].

### *emplk1* is specifically expressed in germinative cells

**Figure 3:**
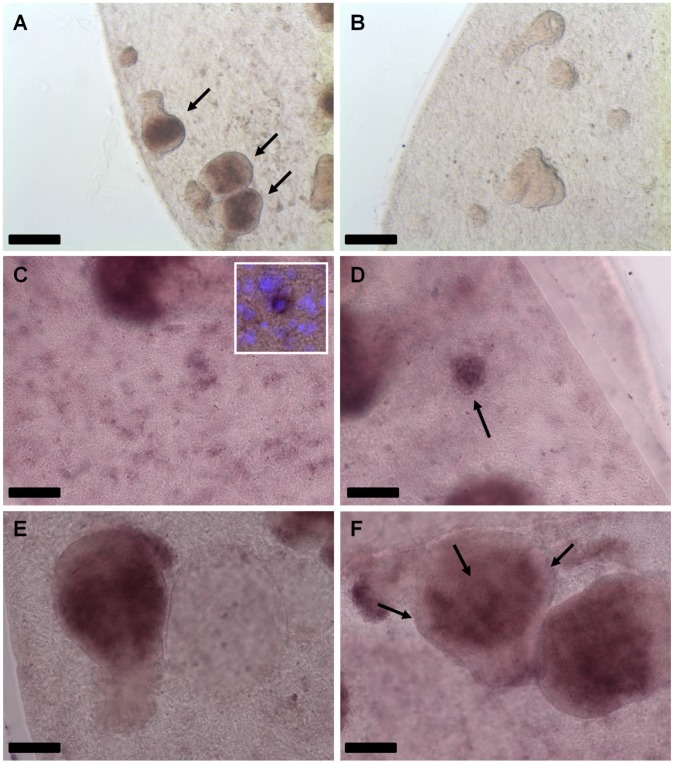
WISH detection of *emplk1* transcripts. (A) General view of a metacestode hybridized with the *emplk1* antisense probe. Note the accumulation of *emplk1* positive cells in the developing protoscoleces (arrows). (B) General view of a metacestode hybridized with the *emplk1* sense control probe. No signal is detected. (C–F) Details of *emplk1* WISH detection in different developmental stages. (C) Detail of the metacestode germinative layer, in which dispersed *emplk1* positive cells can be distinguished (inset: higher magnification of a positive cell, combined with 4′,6-diamidino-2-phenylindole (DAPI) nuclear staining). (D) Accumulation of *emplk1* positive cells in the early brood capsule bud (arrow). (E) Early protoscolex development, with abundant *emplk1* positive cells in the interior. (F) Late protoscolex development. *emplk1* positive cells accumulate at the base of the developing suckers (arrows). Bars represent 200 µm (A, B) and 50 µm (C–F).

We recently investigated cellular proliferation profiles in *E. multilocularis* development [14] and demonstrated that mitotically active parasite stem cells are distributed throughout the germinal layer, and are strongly accumulated in brood capsule and protoscolex buds. In late stage protoscoleces, stem cells are prominently located at the base of developing suckers, but are also present in the posterior body [14]. To investigate a possible stem cell specific expression of *emplk1*, we therefore carried out *in situ* hybridization experiments using a recently established protocol that is applicable to metacestode vesicles [14]. As depicted in Figure 3, prominent *emplk1* signals were obtained for all regions of parasite larvae that typically contain large numbers of proliferating stem cells, such as early brood capsules (Figure 3D), and developing protoscoleces (Figure 3E,F). Furthermore, germinal layer cells with a distribution highly reminiscent of germinative stem cells stained positive for *emplk1* (Figure 3C). Taken together, these data strongly indicated that *emplk1* is specifically expressed in parasite stem cells.

### EmPlk1 is enzymatically active

For functional studies on the schistosome PLK SmPlk1, the heterologous *Xenopus* oocyte expression system has previously been employed [21], and in the present study we used this system to further characterize EmPlk1. To this end, we first generated a mutant form of EmPlk1 in which T_179_ of the activation loop was replaced by phospho-mimetic aspartate (T_179_D), thus yielding a constitutively active form of the enzyme (similar to [21]). We also produced a mutant in which T_179_ was replaced by valine (T_179_V), which would prevent phosphorylation, and thus activation, at T_179_ in *Xenopus* oocytes. Finally, we produced ‘kinase dead’ versions by replacing the D_163_FG motif of the active loop by D_163_SV in both the wild-type and the T_179_D background (wt^KD^; T_179_D^KD^). In *Xenopus* oocytes, it has already been demonstrated that the injection of mRNA encoding activated forms of Plx1 (the Xenopus Plk1-like kinase) or SmPlk1 can induce meiosis resumption, which results in germinal-vesicle breakdown (GVBD) [21]. We therefore injected mRNAs encoding the wild-type and mutant forms of EmPlk1 into *Xenopus* oocytes and carried out GVBD assays.

As shown in Table 1, injection of (non-activated) wild-type EmPlk1 alone was ineffective in inducing GVBD. However, injection of the (activated) T_179_D mutant led to GVBD in 90% of oocytes, which is comparable to activities previously determined for SmPlk1 in this system [21]. The induction of GVBD by an activated mutant of SmPlk1 in *Xenopus* oocytes has previously been shown to involve increased phosphorylation of *Xenopus* Cdc25C [21] and, although we did not specifically test this for EmPlk1, we assume that the *Echinococcus* enzyme is also able to directly activate the *Xenopus* downstream target. In the case of the kinase dead version of activated EmPlk1 (T_179_D^KD^), on the other hand, only a basal rate (10%) of GVBD was observed (Table 1), indicating that the kinase activity of EmPlk1 is responsible for meiosis resumption in *Xenopus*. The T_179_V version of EmPlk1 was also completely inactive in inducing *Xenopus* oocyte GVBD, indicating that phosphorylation of EmPlk1 at T_179_ is essential for enzymatic activity. It should be noted that BI 2536 was rather ineffective in inhibiting GVBD in response to the positive control progesterone (Table 1). This is in line with previous investigations showing that progesterone-induced GVBD of *Xenopus* oocytes occurs via several pathways, among which are the cAMP pathway (involving protein kinase A) and the mitogen-activated protein kinase (MAPK) pathway (including Mos, an oocyte-specific MAPKKK) [39]. Furthermore, meiosis entry of *Xenopus* oocytes in response to progesterone also depends on a balance between Cyclin B synthesis and the activity of Myt1, a member of the Wee1 family of inhibitory kinases, in a Plx1- and MAPK-independent manner [40]. Hence, although Plx1 is required for the activation of Cdc25C in *Xenopus* oocytes [41], leading to accelerated meiosis resumption, Plx1 inhibition can eventually not prevent GVBD. The fact that even high concentrations of BI 2536 do not significantly affect progesterone-induced GVBD in our experiments thus indicates that this compound is not generally cytotoxic to *Xenopus* oocytes and that the BI 2536 effects on EmPlk1 T_179_D-induced GVBD solely result from the specific inhibition of the parasite enzyme.

**Table 1:** Analysis of EmPlk1 activity in *Xenopus* oocytes.

BI2536	PG	EmPlk1 (wt)	T_179_D	T_179_V	EmPlk1 (wt^KD^)	T_179_D^KD^
0 nM	**90**	**0**	**90**	**0**	**0**	10
1 nM	-	-	**90**	-	-	-
5 nM	-	-	**80**	-	-	0
10 nM	**90**	-	**20**	-	-	-
20 nM	-	-	**10**	-	-	0
50 nM	-	-	**0**	-	-	-
100 nM	**80**	-	**0**	-	-	0

Wild-type (wt) and mutant forms (T_179_D, constitutively active; T_179_V, non-activatable; wt^KD^, T_179_D^KD^, kinase dead mutants) of EmPlk1 were expressed in *Xenopus* oocytes (20 oocytes per set) and germinal vesicle breakdown (GVBD) assays were carried out in the presence of different concentrations of BI 2536 as indicated to the left. Treatment by the natural inducer, progesterone (PG), served as a positive control. Displayed are the percentages of *Xenopus* oocytes that underwent GVBD in the different experimental settings (mean of three independent experiments). ‘-’ indicates that this condition has not been tested.

Taken together, these experiments verified that EmPlk1 is an enzymatically active kinase that can stimulate meiosis resumption in the *Xenopus* oocyte system similarly to SmPlk1.

### EmPlk1 activity is affected by PLK-inhibitors

Having established that EmPlk1 can induce GVBD in *Xenopus* oocytes, we then tested whether an available inhibitor, BI 2536, originally designed against human Plk1 [23], is also able to affect the parasite protein. As shown in Table 1, already at concentrations as low as 5 nM, the capability of EmPlk1 to induce GVBD in *Xenopus* oocytes started to diminish, and was completely abolished at concentrations of 50 nM or higher. Similar results were observed when we used BI 6727, another Plk1-specific inhibitor with improved pharmacokinetic profile [27], although in this case slightly higher concentrations were necessary to induce EmPlk1 inhibition (data not shown). Taken together, these experiments indicated that EmPlk1 can be inhibited by BI 2536 and BI 6727 in a concentration dependent manner.

### BI 2536 affects metacestode development from stem cells

**Figure 4:**
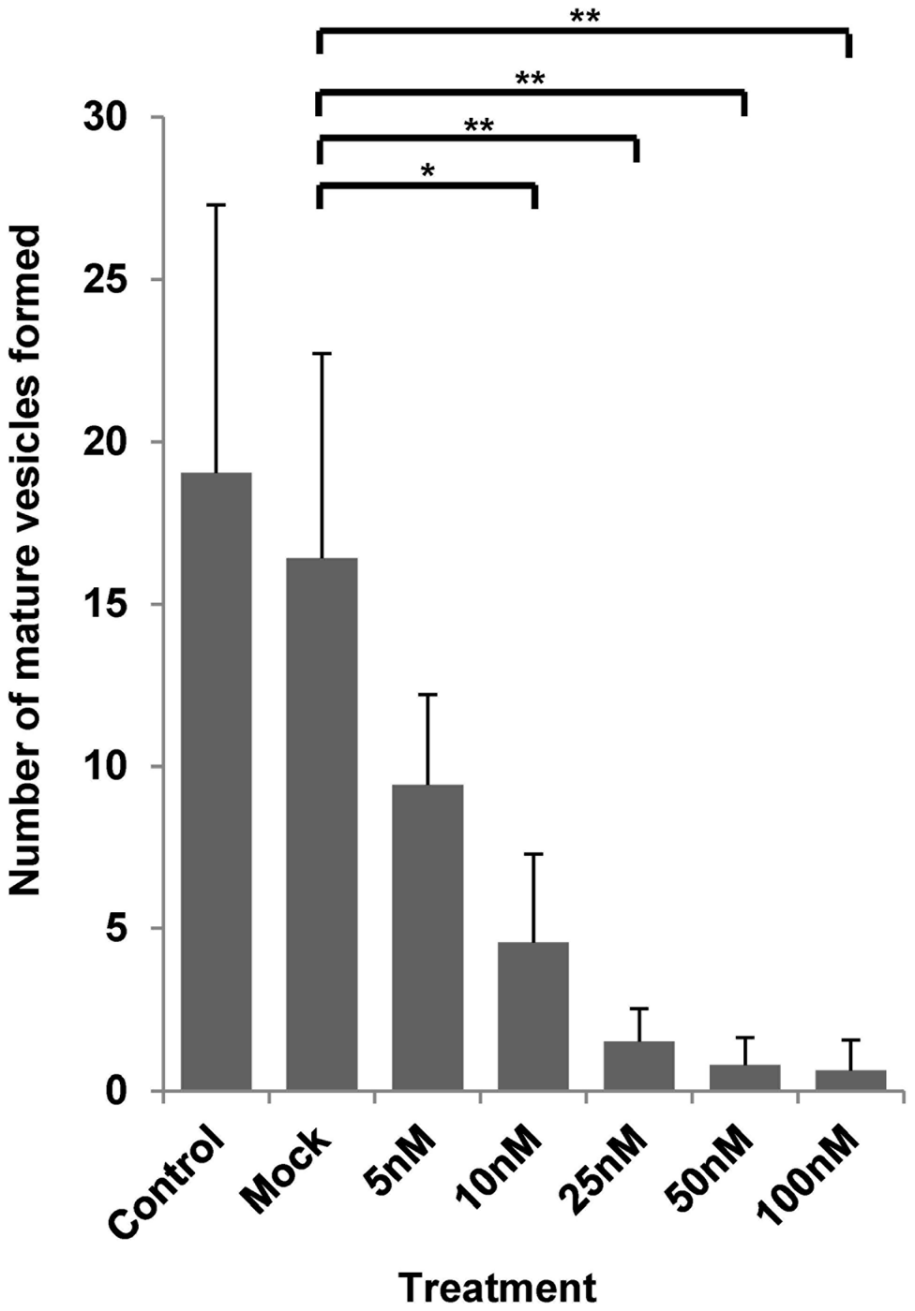
BI 2536 inhibits the formation of metacestode vesicles from *E. multilocularis* primary cells. Primary cell cultures were established from metacestode vesicles and incubated under ideal growth conditions (A4 medium) in the presence of different concentrations of BI 2536 (5, 10, 25, 50, 100 nM) as indicated. After 14 days of incubation, fully formed (mature) metacestode vesicles were counted microscopically. ‘Control’ indicates cultures with A4 medium, ‘mock’ indicates cultures with DMSO (without inhibitor). All conditions were at least assessed in three different biological replicates with at least three technical replicates. *(p<0.05); **(p = 0.001–0.01) (Student's t-test).

Having established that the PLK inhibitor BI 2536 affects the enzymatic activity of EmPlk1, we next investigated whether this compound could also inhibit the formation of metacestode vesicles from parasite stem cells. To this end, we set up *Echinococcus* primary cell cultures and measured the formation of mature metacestode vesicles in the presence of different concentrations of BI 2536. As shown in Figure 4, metacestode formation was already significantly reduced in the presence of 5 nM BI 2536, while concentrations of 25 nM or higher almost completely prevented parasite development. We also tested BI 6727 in the primary cell culture system and, as BI 2536, this inhibitor prevented metacestode vesicle formation in a concentration dependent manner, although at slightly higher concentrations than BI 2536 (data not shown). Since the formation of metacestode vesicles in the primary cell system crucially depends on proliferating stem cells [14], and since we had already shown that EmPlk1 is specifically expressed in this cell type (see above), we therefore concluded that the inhibition of EmPlk1 by BI 2536 and BI 6727 either resulted in stem cell killing, or at least prevented stem cell proliferation, in the primary cell system.

### BI 2536 treatment affects the stem cell system of metacestode vesicles

**Figure 5:**
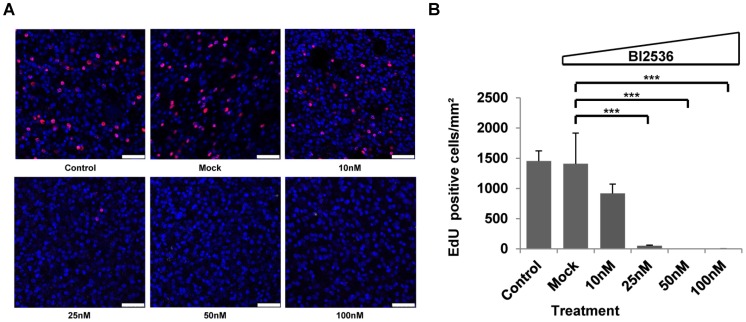
Long-term BI 2536 effects on *E. multilocularis* metacestode vesicles. Metacestode vesicles were treated for 21 days with different concentrations (10, 25, 50 and 100 nM) of BI 2536, followed by three days of recovery without inhibitor. On day three of recovery, EdU labeling (5 h, 50 µM EdU) was carried out and labeled cells were microscopically counted. (A) Microscopic images of the GL of BI 2536 treated (10, 25, 50, 100 nM) and non-treated *E. multilocularis* metacestode vesicles, stained with EdU. Bar represents 25 µm. Blue, nuclear staining, DAPI; red, EdU staining. (B) EdU-positive cell counts per mm^2^ of GL. ‘Control’ indicates vesicles cultivated in A4 medium alone. ‘Mock’ indicates the DMSO control. ***(p<0.001) (Student's t-test). Please note that the specific examples shown in (A) display slightly different total cell numbers but that the overall cell numbers in metacestode vesicles were not altered by drug treatment, as already outlined in [14] for HU treatment.

**Figure 6:**
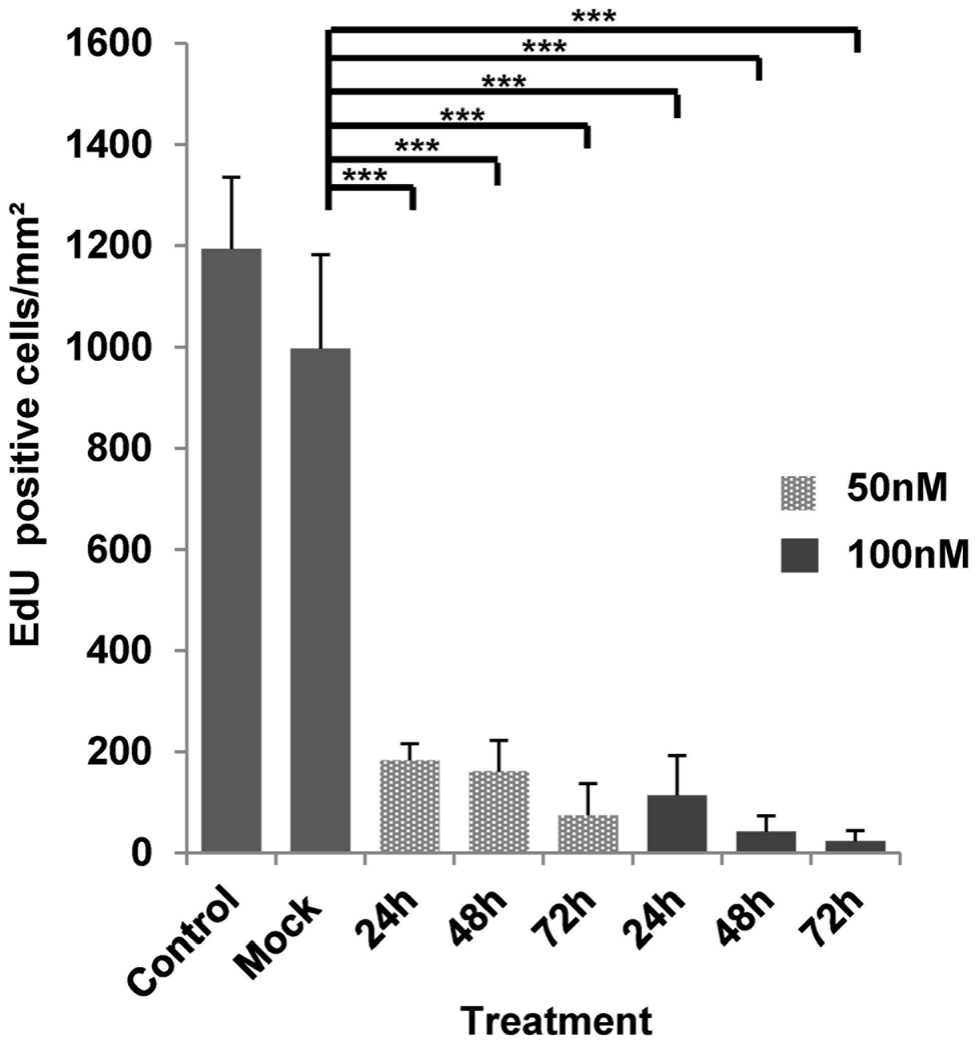
Short-term treatment of metacestode vesicles with BI 2536. Metacestode vesicles were treated for 24, 48 or 72(as indicated) with 50 nM (light grey) or 100 nM (dark grey) of BI 2536, followed by one day of recovery without inhibitor. EdU labeling (5 h, 50 µM EdU) was carried out and labeled cells were microscopically counted. Shown are EdU-positive cell counts per mm^2^ of GL. ‘Control’ indicates vesicles cultivated in A4 medium alone. ‘Mock’ indicates the DMSO control. ***(p<0.001) (Student's t-test).

We then tested the effects of BI 2536 on mature metacestode vesicles, which is the actual target stage of chemotherapy in AE [1], [5]. Interestingly, as shown in Figure S2, even incubation for 21 days in the presence of 100 nM BI 2536 did not lead to structural disintegration or collapse of metacestode vesicles which, however, had slightly reduced sizes and were no longer capable of growing. This was similar to an approach we had recently undertaken concerning metacestode treatment with the ribonucleotide reductase inhibitor hydroxyurea (HU), which is toxic to cells that undergo proliferation [14]. Although HU treatment specifically eliminated germinative cells in metacestode vesicles, these remained structurally intact for several weeks, indicating that this parasite stage is able to survive for long periods under conditions of slow cellular turnover [14]. We therefore tested whether BI 2536 treatment of metacestode vesicles might specifically eliminate the germinative cell population and carried out EdU incorporation experiments. To this end, metacestode vesicles were incubated for 21 days in presence of different concentrations of BI 2536. After recovery for 3 days without inhibitor, proliferating cells were detected by EdU pulse labeling [14]. As shown in Figure 5, control vesicles displayed a typical pattern of proliferating germinative cells within the germinal layer. In samples treated with 10 nM BI 2536, however, the number of proliferating stem cells was reduced by ∼50%. A statistically significant reduction to ∼10% of normal numbers was observed after incubation with 25 nM BI 2536 and in the presence of higher inhibitor concentrations (50, 100 nM), cell proliferation in the germinal layer was completely abolished (Figure 5). Like in our previous experiments using HU as an inhibitor [14], no effects of BI 2536 treatment were observed on differentiated cells. As expected from the absence of proliferating cells, even after 3 weeks of further cultivation, these vesicles did not resume growth or proliferation capacity (data not shown). Using vesicles after 21 day treatment with 50 or 100 nM BI 2536 as a source for parasite primary cell cultivation, we also never obtained cultures that formed typical cell aggregates [13], [14] or mature metacestode vesicles (data not shown). Finally, we also carried out short term treatment (24, 48, 72 h) of metacestode vesicles with 50 nM and 100 nM BI 2536 concentrations, followed by 1 day recovery and 5 h EdU pulses. As shown in Figure 6, already after 24 h in the presence of 50 nM BI 2536, the number of proliferating cells within the germinal layer was reduced to ∼17% of the normal proliferating cell number, and it was further diminished after incubation for 72 h or at higher concentrations (100 nM; Figure 6).

Taken together, the experiments outlined above clearly indicated that BI 2536 specifically targeted the germinative stem cell population of metacestode vesicles and either led to stem cell killing or long-term mitotic arrest.

## Discussion

The pathology of AE is crucially linked to continuous asexual growth of the *E. multilocularis* metacestode stage within the intermediate host's liver, accompanied by metastasis formation in secondary organs [1], [2], [4], [5]. Since somatic stem cells are typically employed for cellular proliferation in flatworms (reviewed in [10]), it has already very early been suggested that a population of undifferentiated cells, which forms part of the *Echinococcus* GL, is responsible for both tumor-like, infiltrative growth of parasite larvae within host organs, and for metastases formation after distribution through the lymphatic system of the host [11], [12], [42]. We previously developed methods to isolate and cultivate these cells, and showed that they are capable of producing new metacestode tissue when kept in culture in the presence of host-derived feeder cells [13]. Very recently, we also showed that these cells exhibit a typical stem cell character and that subpopulations of these cells express several genes of the *nanos* and *argonaute* family that are typical components of the germline multipotency program of metazoan stem cells [14]. We also demonstrated that this cell type, called ‘germinative cells’, is the only cell type capable of proliferation in *E. multilocularis* larvae and that it produces all differentiated cell types present in the metacestode [14]. Since germinative cells are capable of producing new metacestode tissue even when removed from their normal tissue context in the germinal layer [13], this cell type thus constitutes a crucial target for the development of anti-*Echinococcus* drugs that aim to prevent parasite proliferation and metastasis formation.

For decades, anti-AE chemotherapy has relied on benzimidazoles (mostly albendazole) [5], and although other compounds with anti-parasitic activities are currently subject to intense research [8], [9], [43], no reliable alternative to benzimidazole treatment is currently in the pipeline. Unfortunately, little information is available concerning direct effects of benzimidazoles on *Echinococcus* germinative cells. However, when we use albendazole in cell killing assays on freshly isolated primary cell cultures, which contain up to 80% germinative cells [14], little or no effects are detected, even at high drug concentrations (Hemer, Brehm, unpublished results). Indications for limited activity of benzimidazoles on parasite stem cells were also obtained in earlier studies by Ingold et al. [44] and Stettler et al. [45] who found that albendazole derivatives only affected the germinative cells (called ‘undifferentiated cells’ in these publications) at late time points of *in vitro* treatment of metacestode vesicles, and much less than other compounds such as nitazoxanide. These *in vitro* studies were recently verified *in vivo* by Küster et al. [46]. The fast recurrence of parasite growth in patients after discontinuation of albendazole chemotherapy [1], [5], [8], which has to rely on surviving germinative cells [14], could therefore be due to limited activities of benzimidazoles against this particular cell type. The molecular basis for this limited efficacy could be stem cell-specific expression of parasite β-tubulin isoforms that are resistant to inhibition by benzimidazoles [7]. We have previously characterized three *E. multilocularis* β-tubulin isoforms of which one, Tub-2, displayed amino acid sequence motifs that indicated limited interaction with benzimidazoles [6] and in transcriptome analyses collected during the *E. multilocularis* genome project [28], the Tub-2 encoding gene (*tub-2*) displayed highest expression in parasite stages that are enriched in germinative cells (Figure S3). Furthermore, in preliminary transcriptome analyses on *E. multilocularis* larvae (unpublished results), we already obtained evidence for stem cell-specific expression of *tub-2*. Hence, apart from limited bioavailability of benzimidazoles at the site of infection [8] and adverse side effects due to high homologies between host- and parasite β-tubulin [6], one of the drawbacks of benzimidazole chemotherapy could be limited efficacy against germinative cells since these express a potentially resistant β-tubulin isoform.

In the present work, we present information on a druggable enzyme that fulfils a crucial role in *Echinococcus* germinative cell proliferation. Our structural analyses clearly identified EmPlk1 as a member of the Plk1-like subfamily of PLKs, with all protein domains and catalytic residues that are typical for this enzyme family at the corresponding positions. By heterologous expression in *Xenopus* oocytes, we also demonstrated that EmPlk1 is an active kinase that can induce meiosis resumption and GVBD. RT-PCR analyses, transcriptome data, and *in situ* hybridization experiments further indicated that EmPlk1 is specifically expressed in *Echinococcus* germinative cells that are present in the germinal layer of the metacestode and in developing protoscoleces. The stem cell-specific expression of EmPlk1 is further supported by preliminary deep sequencing transcriptome data of our group which show that HU treated, and thus stem cell depleted, metacestode vesicles [14] are dramatically reduced in *emplk1* transcripts when compared to untreated metacestode vesicles (unpublished data). Based on these data and on the conserved functions of Plk1-like kinases in other metazoans [15]–[18], we propose that EmPlk1 fulfils an important function in *Echinococcus* germinative cells, particularly in dividing stem cells during G2/M phase transition. An important upstream interaction partner of human Plk1 is the kinase Aurora A, which phosphorylates, and thus activates, Plk1 at T_210_ [15]. Since we have shown that EmPlk1 also requires activation at a corresponding threonine residue (T_179_) we propose that a similar activation mechanism also exists in *Echinococcus* cells, and according to the genome sequence [28], the parasite indeed encodes a gene encoding an Aurora A-like kinase (EmuJ_001059700). Important downstream factors for human Plk1 are the tyrosine phosphatase Cdc25c (also called M-phase inducer phosphatase) which directs dephosphorylation of cyclin B-bound CDK1 (cyclin-dependent kinase 1), thus triggering entry into mitosis [15], the transcription factor forkhead box M1 (FoxM1), which regulates the expression of a cluster of G2/M target genes, and the tumor suppressor p53 [15], [16]. Orthologs to these factors are also present in the *Echinococcus* genome, such as an M-phase inducer phosphatase gene (EmuJ_001174300), a p53 ortholog (annotated as p63; EmuJ_000098700), and several genes encoding forkhead transcription factors (e.g. EmuJ_000620400). Notably, according to preliminary transcriptome data (unpublished results), the genes encoding homologs to Aurora A, p53, and several forkhead transcription factor genes are expressed in a germinative cell-specific manner and could thus, together with *emplk1*, form a regulatory network that controls the *Echinococcus* stem cell cycle similar to the situation in humans [15], [16].

In *S. mansoni*, Long et al. [22] recently characterized a Plk4-like PLK, named SmSak, which interacts with SmPlk1 in *Xenopus* oocytes, and which is co-expressed with SmPlk1 in the female ovary and vitellarium. These authors showed that SmSak can be activated following its interaction with SmPlk1, indicating a potential role of SmSak in schistosome meiosis. SmSak was not, however, inhibited by PLK-inhibitors directed against Plk1-like family members. Together with their previous characterization of SmPlk1 [21], these authors thus demonstrated that schistosomes employ an invertebrate typical set of PLKs, consisting of one member of the Plk1 and one member of the Plk4 sub-families. In our BLASTP analyses, we also noted the presence of gene encoding a second PLK in *E. multilocularis* (EmuJ_000104700). However, although the kinase domain of the encoded protein displayed similarity to PLK kinase domains, overall sequence similarity of this protein was highest with Plk4 subfamily members (including SmSak) and the protein obviously lacked conserved C-terminal PBDs (data not shown). Of the 8 amino acid residues known to be involved in the binding of Plk1 inhibitors (such as BI 2536) to human Plk1 [38], which were all conserved in EmPlk1, only one was conserved in the kinase domain of this putative EmSak. In the position corresponding to L_101_ of EmPlk1, which in human Plk1 determines BI 2536 specificity [38], the kinase domain of this protein contains a bulky residue (phenylalanine). We therefore believe that the protein encoded by EmuJ_000104700 fulfils similar activities as SmSak, particularly in centriole duplication [22], and might even interact with EmPlk1, but that none of the activities of BI 2536 discussed below are due to inhibition of the kinase activity of this protein.

Due to its overexpression in many human tumors, Plk1 has been extensively studied as a target for anti-cancer therapy and a number of compounds that either act as ATP-competitive inhibitors or interfere with Polo-box domain functions of Plk1 have already been identified [15], [18]. One of the best studied compounds in this regard is the ATP-competitive inhibitor BI 2536, which causes apoptosis and prometaphase arrest in a variety of tumor cell lines [15], [18], [47], [48]. In several phase I and phase II clinical trials against lung or pancreatic cancer, BI 2536 was well tolerated, albeit with varying success rates, depending on the nature of the tumor [15], [18], [23], [48]–[52]. In *in vitro* studies, BI 2536 usually displays activities against human tumor cell lines in an IC_50_ range between 5 and 175 nM [15], [18], [47], [48] and, depending on the intravenous doses given, tolerable plasma concentrations of BI 2536 in clinical trials on cancer patients vary between 20 and 200 nM [23], [49]–[52]. This is well within the range of BI 2536 activities that we observed herein against EmPlk1 and *E. multilocularis* larvae. As we have shown, concentrations as low as 20 nM BI 2536 almost completely inhibited the activity of EmPlk1 in *Xenopus* oocytes, and at higher concentrations (50, 100 nM), EmPlk1 was no longer able to induce GVBD. Since all amino acid residues previously determined to mediate binding of BI 2536 to human Plk1 are also highly conserved in EmPlk1, it is reasonable to assume that BI 2536-mediated inhibition of both enzymes follows a similar ATP-competitive mechanism. We further demonstrated that BI 2536 concentrations of 25 nM and higher were very effective in preventing metacestode vesicle formation from parasite germinative cells, and in depleting metacestode vesicles of germinative cells. The specific elimination of germinative cells in metacestode vesicles, which otherwise remained intact for several weeks, is yet another indicator for stem cell specific expression of EmPlk1, and we propose that the BI 2536 effects we observed in the primary cell system are also due to EmPlk1 inhibition in the stem cell population. It is not yet clear whether BI 2536 treatment of parasite larvae induces germinative cell killing or just (transient) mitotic arrest. However, even weeks after treating metacestode vesicles with 50 or 100 nM BI 2536, we never observed growth resumption or a re-population of the GL with germinative cells. Furthermore, it was not possible to set up proliferating parasite primary cell cultures from vesicles that had been treated with 50 or 100 nM BI 2536. Together with observations that BI 2536 can induce apoptosis and severe phenotypes in human cancer cells [15], [18], we therefore hypothesize that BI 2536 treatment of *E. multilocularis* indeed led to germinative cell killing, or at least to permanent mitotic arrest.

Taken together, we herein present a promising target for the development of anti-echinococcosis drugs that specifically affect the germinative (stem) cell system of the parasite and, thus, would ideally complement anti-parasitic activities of benzimidazoles. On the one hand, BI 2536 itself could already be administered to infected mice, combined with benzimidazoles, to study possible additive effects. Respective experiments are currently planned in our laboratory. Furthermore, BI 2536 could serve as a lead compound for the identification of drugs that are more specific to the parasite Plk1 when compared to human Plk1. Although the kinase domains of both enzymes are homologous (62% identical residues), they are clearly more divergent than are host and parasite β-tubulins (>90% [6]), and should contain structural differences that can be exploited for parasite-specific drug design. This possibility is supported by the fact that the second generation Plk1 inhibitor BI 6727, which has similar affinities and a similar binding mode to human Plk1 as BI 2536 [27], was less effective in preventing metacestode formation and in inhibiting EmPlk1 in the *Xenopus* system than BI 2536 and also shows 4- and 11-fold less selectivity against human Plk2 and Plk3, respectively, than BI 2536 [27]. Despite these somewhat lower activities of BI 6727 in eliminating *Echinococcus* stem cells *in vitro*, it should not, however, be dismissed as a potential anti-echinococcosis drug due to its clearly improved pharmacokinetic profile and the fact that it can be given orally [27]. Finally, our characterization of EmPlk1 as a factor that governs the mitotic activity of *E. multilocularis* germinative cells will form a solid basis for further investigations into the regulation of the unique stem cell system of this parasite.

## Supporting Information

Figure S1 **Transcriptomic analysis of** ***emplk1*** **expression in** ***E. multilocularis*** **larvae.** Illumina transcriptome sequencing has been carried out for some life cycle stages during the *E. multilocularis* genome project [28]. Shown are fpkm (fragments per kilobase of exon per million fragments mapped) values for *emplk1* for primary cells after 2 (PC2) and 11 days (PC11) of development as well as metacestode vesicles (MC) and dormant (PS−) and pepsin/low Ph-activated (PS+) protoscoleces. Note that Illumina sequencing has been performed only once for each sample.
(TIF)

Figure S2 **Effects of BI 2536 on metacestode vesicle integrity.** Metacestode vesicles were treated for 21 days with 100 nM Bi 2536 and vesicle integrity was visually inspected. (A) Number of structurally integer vesicles after treatment. (B) Culture flasks showing vesicles after 21 day treatment (BI 2536, 100 nM) and control vesicles. Note that the BI 2536 treated vesicles are smaller and darker in appearance, but are still round and floating.
(TIF)

Figure S3 **Transcriptomic analysis of β-tubulin gene expression in** ***E. multilocularis*** **larvae.** Illumina transcriptome sequencing has been carried out for some life cycle stages during the *E. multilocularis* genome project [28]. Shown are fpkm (fragments per kilobase of exon per million fragments mapped) values for the genes *tub-1*, *tub-2*, and *tub-3* as indicated to the right. Shown are values for primary cells after 2 (PC2) and 11 days (PC11) of development as well as metacestode vesicles (MC) and dormant (PS−) and pepsin/low Ph-activated (PS+) protoscoleces. Note that Illumina sequencing has been performed only once for each sample.
(TIF)
