# Supplementary figures and images for: Targeting Echinococcus multilocularis Stem Cells by Inhibition of the Polo-Like Kinase EmPlk1
Source: PLoS Negl Trop Dis. 2014 Jun 5;8(6):e2870. doi: 10.1371/journal.pntd.0002870 (PMC4046951; doi:10.1371/journal.pntd.0002870)

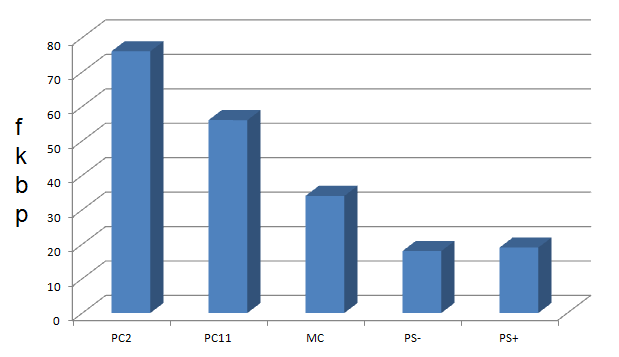

Supplement: Figure S1 — Transcriptomic analysis of emplk1 expression in E. multilocularis larvae. Illumina transcriptome sequencing has been carried out for some life cycle stages during the E. multilocularis genome project [28]. Shown are fpkm (fragments per kilobase of exon per million fragments mapped) values for emplk1 for primary cells after 2 (PC2) and 11 days (PC11) of development as well as metacestode vesicles (MC) and dormant (PS−) and pepsin/low Ph-activated (PS+) protoscoleces. Note that Illumina sequencing has been performed only once for each sample. (TIF) [file pntd.0002870.s001.tif]

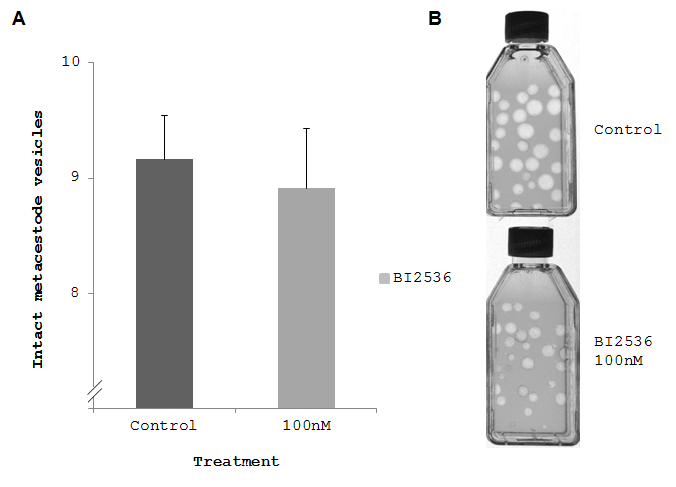

Supplement: Figure S2 — Effects of BI 2536 on metacestode vesicle integrity. Metacestode vesicles were treated for 21 days with 100 nM Bi 2536 and vesicle integrity was visually inspected. (A) Number of structurally integer vesicles after treatment. (B) Culture flasks showing vesicles after 21 day treatment (BI 2536, 100 nM) and control vesicles. Note that the BI 2536 treated vesicles are smaller and darker in appearance, but are still round and floating. (TIF) [file pntd.0002870.s002.tif]

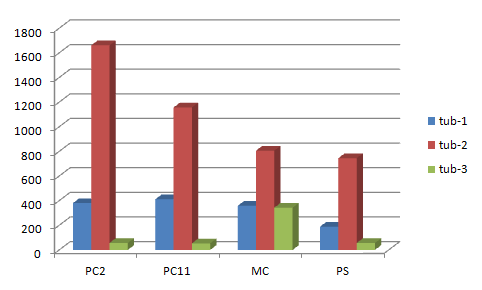

Supplement: Figure S3 — Transcriptomic analysis of β-tubulin gene expression in E. multilocularis larvae. Illumina transcriptome sequencing has been carried out for some life cycle stages during the E. multilocularis genome project [28]. Shown are fpkm (fragments per kilobase of exon per million fragments mapped) values for the genes tub-1, tub-2, and tub-3 as indicated to the right. Shown are values for primary cells after 2 (PC2) and 11 days (PC11) of development as well as metacestode vesicles (MC) and dormant (PS−) and pepsin/low Ph-activated (PS+) protoscoleces. Note that Illumina sequencing has been performed only once for each sample. (TIF) [file pntd.0002870.s003.tif]
